# Eradication therapy may decrease the risk of immune thrombocytopenia after *Helicobacter pylori* infection: a retrospective cohort study in Taiwan

**DOI:** 10.1186/s12876-023-02664-z

**Published:** 2023-02-08

**Authors:** Mitsuhiro Koseki, Ming‑Jen Sheu, Kang-Ting Tsai, Chung-Han Ho, Hsiao-Hua Liu, Hung-Jung Lin, Chien-Liang Lin, Chien-Cheng Huang

**Affiliations:** 1 Department of Internal Medicine, Chi Mei Medical Center, Tainan, Taiwan; 2 Division of Gastroenterology and Hepatology, Department of Internal Medicine, Chi Mei Medical Center, Tainan, Taiwan; 3 Division of Geriatrics and Gerontology, Department of Internal Medicine, Chi Mei Medical Center, Tainan, Taiwan; 4 Department of Senior Services, Southern Taiwan University of Science and Technology, Tainan, Taiwan; 5 Department of Medical Research, Chi Mei Medical Center, Tainan, Taiwan; 6 Department of Information Management, Southern Taiwan University of Science and Technology, Tainan, Taiwan; 7 Department of Emergency Medicine, Chi Mei Medical Center, 901 Zhonghua Road, Yongkang District, Tainan City, 710 Taiwan; 8 Department of Emergency Medicine, Taipei Medical University, Taipei, Taiwan; 9 Division of Hematology-Oncology, Department of Internal Medicine, Chi Mei Hospital, 201, Taikang, Liouying District, Tainan City, 736 Taiwan; 10 Department of Emergency Medicine, Kaohsiung Medical University, Kaohsiung, Taiwan

**Keywords:** Adult, Cohort study, Eradication therapy, *Helicobacter pylori*, Immune thrombocytopenia

## Abstract

**Background:**

*Helicobacter pylori* (HP) eradication therapy (HPE) is recommended for patients with unexplained immune thrombocytopenia (ITP); however, the role of HPE in preventing ITP in patients with HP infection remains unclear. Therefore, this study was designed to clarify it.

**Methods:**

This study was conducted at a tertiary medical center and included all adult patients with HP infection between January 1, 2016 and December 31, 2018. We compared the risk of developing ITP between patients with and without HPE. All patients were followed up until December 31, 2020.

**Results:**

After excluding patients with thrombocytopenia, 1995 adult patients with HP infection, including 1188 patients with HPE and 807 patients without HPE, were included in this study. The mean age of the patients with HPE was 57.9 years, whereas that of those without HPE was 61.6 years. The percentage of males was 56% in patients with HPE and 59% in those without HPE. Patients without HPE had a higher risk of ITP than those with HPE after adjusting for age, sex, the Charlson Comorbidity Index, and comorbidities [adjusted odds ratio (OR) 1.76; 95% confidence interval (CI) 1.16–2.68]. Stratified analyses showed that the higher risk was found only in males (adjusted OR: 1.70; 95% CI 1.03–2.80). In addition to HPE, male sex and anemia were independent predictors of ITP in patients with HP infection.

**Conclusion:**

This study showed that adult patients with HP infection not receiving HPE had a higher risk of developing ITP. We suggest that HPE should be considered, particularly in males and those who have anemia, to prevent ITP.

**Supplementary Information:**

The online version contains supplementary material available at 10.1186/s12876-023-02664-z.

## Introduction

*Helicobacter pylori* (HP) is a common and important infective microorganism worldwide, which may contribute to gastritis, peptic ulcer disease, unexplained iron deficiency anemia, gastric atrophy, mucosal-associated lymphoid tissue lymphoma, and gastric cancer [1]. It is estimated that more than half of the world’s population is infected by HP [2]. The prevalence of HP infection varies greatly across countries, with Africa (70.1%) having the highest prevalence, followed by South America (69.4%), Taiwan (53.9%), and Japan (51.7%), and Oceania (24.4%) having the lowest prevalence [2]. Immune thrombocytopenia (ITP) is an autoimmune disease characterized by isolated thrombocytopenia [3]. The major complications of ITP are severe bleeding in 15% of patients and affected patients have twice the risk of venous thromboembolism compared with the general population [3].

Studies showed that HP infection may be associated with ITP, and therefore, HP eradication therapy (HPE) is recommended for patients with unexplained ITP [4]. Some studies even proposed that HPE was the first-line treatment for chronic ITP [5, 6]. All studies focused on HPE for treating existing ITP. To the best of our knowledge, no study focused on the effects of HPE on patients with HP infection without thrombocytopenia. Therefore, this study was designed to clarify whether HPE can prevent ITP in patients with HP infection.

## Materials and methods

### Study design, setting, and patients

**Fig. 1:**
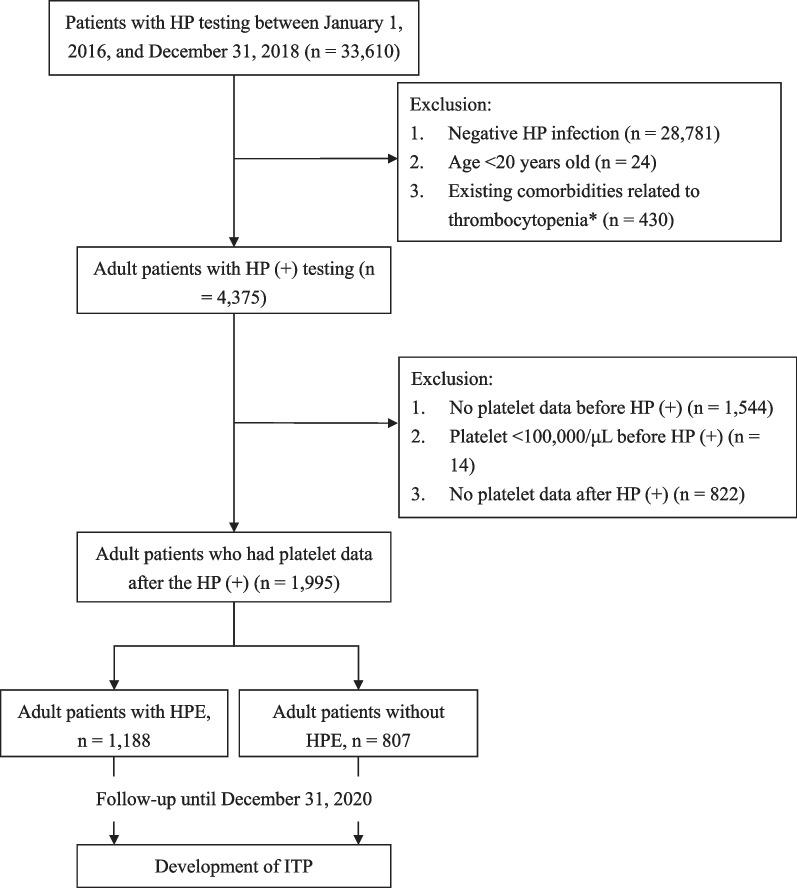
Identification and exclusion of the patients. HP, *Helicobacter pylori*; HPE, *Helicobacter pylori* eradication therapy. *Other possible diseases that may cause thrombocytopenia: hepatitis B, hepatitis C, tuberculosis, human immunodeficiency virus disease, hyperthyroidism, hypothyroidism, thyroiditis, simple and unspecified goiter, non-toxic nodular goiter, thyroid diseases, neoplasms, hematological malignancies, carcinoma in situ, neoplasm of uncertain behavior of other lymphatic and hematopoietic tissues, hemorrhagic disorder due to circulating anticoagulants, immunodeficiency, macroglobulinemia, sarcoidosis, rheumatoid arthritis, systemic lupus erythematosus, polymyositis, dermatomyositis, sicca syndrome, systemic sclerosis, vasculitis, antiphospholipid syndrome (abnormal of anti-cardiolipin IgG, B2-Glycoprotein I IgG, and lupus anticoagulant test), and other autoimmune or diffuse connective tissue diseases [4, 8–10]

This study was conducted at the Chi Mei Medical Center (CMMC), a tertiary medical center in Southern Taiwan [7]. First, we identified all patients who underwent HP testing at the CMMC between January 1, 2016 and December 31, 2018 for this study (Fig. 1). Second, the exclusion criteria were as follows: (1) patients with negative HP infection; (2) those aged < 20 years old; or (3) those with existing comorbidities related to thrombocytopenia, including hepatitis B, hepatitis C, tuberculosis, human immunodeficiency virus disease, thyroid diseases, neoplasms, hematological malignancies, carcinoma in situ, hemorrhagic disorder due to circulating anticoagulants, immunodeficiency, macroglobulinemia, sarcoidosis, rheumatoid arthritis, systemic lupus erythematosus, polymyositis, dermatomyositis, sicca syndrome, systemic sclerosis, vasculitis, antiphospholipid syndrome (abnormal of anti-cardiolipin IgG, B2-Glycoprotein I IgG, and lupus anticoagulant test), and other autoimmune or diffuse connective tissue diseases [4, 8–10]. We excluded patients aged < 20 years because we wanted to study adult patients and the adult age was set at 20 years during the study period in Taiwan [11]. The diagnosis of HP infection was made using either the rapid urease test or biopsy with panendoscopy [12]. Third, we excluded patients who had the following criteria: (1) no platelet data before HP (+); (2) platelet < 100,000/μL before HP (+); or (3) no platelet data after HP (+).

### Two cohorts: patients with HPE versus patients without HPE

The final identified adult patients with HP infection were divided into patients with HPE and patients without HPE. The standard HPE in the CMMC included antibiotics and proton pump inhibitors, according to the consensus in Taiwan [13]. Patients who had received those drugs beyond 7 days were defined as the study cohort [13]. Others who did not complete the treatment were defined as the comparison cohort. The antibiotics for HPE were clarithromycin, amoxicillin, tetracycline, and metronidazole [13]. The proton pump inhibitors were pantoprazole, dexlansoprazole, esomeprazole, lansoprazole, omeprazole, and rabeprazole. Some patients who had received bismuth subsalicylate were also included.

### Variables and data collection

We collected data, including demographic characteristics, comorbidities, the Charlson Comorbidity Index (CCI), and laboratory data, from the electronic medical records at the CMMC for analysis. The comorbidities were diagnosed by the treating physicians, including peptic ulcer disease, hypertension, gastroesophageal reflux disease (GERD), diabetes, stroke, anemia, and coronary artery disease. The comorbidities were counted at the time that the patient was diagnosed with HP infection. Data collection was performed by an experienced researcher who was blinded to the outcomes of the patients. The study was unaffected by coronavirus disease 2019 because there was no pandemic outbreak in Taiwan during the study period. The CCI score was divided into three subgroups: 0, 1–2, and ≥ 3 [14].

### Outcome measurements

We compared the risk of developing ITP between the two cohorts by following them up until December 31, 2020. The platelet count was checked every six months during the follow-up. The definition of ITP was as follows: (1) platelets < 100,000 per μL; (2) exclusion of other possible causes of thrombocytopenia; and (3) erythrocytes and leukocytes are within the normal range [8]. The risk of developing severe thrombocytopenia (platelets < 30,000 per μL) [15] between the two cohorts was also investigated. We also analyzed the patients with platelet count between 100,000 and 150,000 per μL for developing ITP, which may be considered an evolving immune patients [16].

### Ethical statements

This study was conducted strictly according to the Declaration of Helsinki and approved by the Institutional Review Board (IRB) of the CMMC (IRB serial no. 11103-002). All patients’ data were anonymized. Patient informed consent was waived because of the retrospective and observational nature of the study by the IRB of the CMMC. The welfare of the patients was unaffected by the waiver.

### Statistics

We used Pearson’s chi-square test for comparing categorical variables and Student’s t-test for comparing continuous variables. The categorical variables were described as frequencies with percentages. The continuous variables were expressed as means ± standard deviations. Multivariate logistic regression analyses were performed to investigate the risk of developing ITP between the two cohorts. Stratified analyses of sex, age, and comorbidities were performed to investigate the effect modification. Furthermore, we performed multivariate logistic regression analyses to identify independent predictors of ITP in the patients with HP infection. All analyses were performed using SAS, version 9.4 (SAS Institute, Inc., Cary, NC, USA). *p*-values of less than 0.05 were used to denote statistical significance.

## Results

In this study, 1,995 adult patients were included, including 1,188 patients with HPE and 807 patients without HPE (Table 1). The male sex predominated both cohorts (56.0% in patients with HPE vs*.* 59.0% in patients without HPE; *p* = 0.183). Patients with HPE were younger than those without HPE (57.9 ± 14.0 years vs*.* 61.6 ± 14.4 years; *p* < 0.001). The patients with HPE had a higher prevalence of peptic ulcer disease and GERD than those without HPE; however, they had a lower prevalence of diabetes and stroke. The CCI score was lower in patients with HPE than in those without HPE.

**Table 1:** Comparison of the baseline characteristics between adult patients with and without HPE by univariate analysis

Variables	With HPE (n = 1,188)	Without HPE (n = 807)	*p*-value*
Sex, n (%)			
Male	665 (56.0)	476 (59.0)	0.183
Female	523 (44.0)	331 (41.0)	
Age, mean ± SD Age subgroup, n (%)	57.9 ± 14.0	61.6 ± 14.4	< 0.001
Age < 65	809 (68.1)	471 (58.4)	< 0.001
Age ≥ 65	379 (31.9)	336 (41.6)	
Comorbidity, n (%)			
Peptic ulcer disease	492 (41.4)	156 (19.3)	< 0.001
Hypertension	295 (24.8)	218 (27.0)	0.274
GERD	230 (19.4)	84 (10.4)	< 0.001
Diabetes	177 (14.9)	188 (23.3)	< 0.001
Stroke	49 (4.1)	74 (9.2)	< 0.001
Anemia	27 (2.3)	24 (3.0)	0.330
Coronary artery disease	6 (0.5)	5 (0.6)	0.765
CCI score, mean ± SD	1.4 ± 1.3	1.7 ± 1.8	< 0.001
CCI subgroup, n (%)			
0	253 (21.3)	249 (30.9)	< 0.001
1–2	755 (63.6)	355 (44.0)	
≥ 3	180 (15.2)	203 (25.2)	

*HPE Helicobacter pylori* eradication therapy, *SD* Standard deviation, *GERD* Gastroesophageal reflux disease, *CAD* Coronary artery disease, *CCI* Charlson comorbidity index

*Categorical variables analysis using Fisher’s exact test and continuous variables analysis using the Mann–Whitney U test

Patients without HPE had a higher risk of developing ITP than those with HPE after adjusting for age, sex, the CCI score, and comorbidities, including peptic ulcer disease, hypertension, GERD, diabetes, stroke, and anemia [adjusted odds ratio (OR):1.76; 95% confidence interval (CI) 1.16–2.68; *p* = 0.008] (Table 2). Stratified analyses showed that male patients without HPE also had a higher risk of ITP (adjusted OR: 1.70; 95% CI 1.03–2.80; *p* = 0.039). However, in the female population, the difference in the risk of developing ITP between the two cohorts was not statistically significant (adjusted OR: 1.69; 95% CI 0.78–3.69; *p* = 0.184).

**Table 2:** Comparison of the risk of ITP between adult patients without and with HPE by multivariate logistic regression analyses

Without HPE versus with HPE (reference)	Patients n	ITP n (%)	Adjusted OR*	*p*-value
Overall analysis	807	70 (8.7)	1.76 (1.16–2.68)	0.008
*Stratified analysis*				
Sex				
Male	476	51 (10.7)	1.70 (1.03–2.80)	0.039
Female	331	19 (5.7)	1.69 (0.78–3.69)	0.184
Age subgroup				
Age < 65	471	36 (7.6)	1.78 (1.00–3.15)	0.050
Age ≥ 65	336	34 (10.1)	1.71 (0.93–3.14)	0.083
Comorbidity				
Peptic ulcer disease	224	17 (7.6)	1.92 (0.96–3.81)	0.064
Hypertension	218	21 (9.6)	1.84 (0.85–3.97)	0.120
GERD	84	4 (4.8)	1.97 (0.38–10.36)	0.423
Diabetes	188	16 (8.5)	1.06 (0.44–2.58)	0.890
Stroke	74	5 (6.8)	2.90 (0.43–19.47)	0.274
Anemia	24	6 (25.0)	3.25 (0.40–26.73)	0.273

*ITP* Immune thrombocytopenia, *HPE Helicobacter pylori* eradication therapy, *OR* Odds ratio, *CCI* Charlson comorbidity index, *GERD* Gastroesophageal reflux disease

*Adjusted for age, sex, the CCI score, and comorbidities, including peptic ulcer disease, hypertension, GERD, diabetes, stroke, and anemia

In addition to HPE, the male sex (adjusted OR: 2.23; 95% CI 1.45–3.43; *p* < 0.001) and anemia (adjusted OR: 3.76; 95% CI 1.69–8.38; *p* = 0.001) were independent predictors of ITP in all adult patients with HP infection (Table 3). Patients without HPE have a higher risk of having a platelet count < 30,000 per μL than those with HPE, after adjusting for age, sex, the CCI score, and comorbidities, including peptic ulcer disease, hypertension, GERD, diabetes, stroke, and anemia (adjusted OR: 8.46; 95% CI 1.74–41.16; *p* = 0.008) (Additional file 1: Table S1). There were 56 patients with platelet count between 100,000 and 150,000 per μL, including 28 patients with HPE and 28 patients without HPE (Additional file 1: Table S2). Compared with patients without HPE, the risk of developing ITP in the patients with HPE was lower; however, the difference was not statistically significant (47.1% vs*.* 52.9%; *p* = 0.771).

**Table 3:** Independent predictors of ITP in all adult patients with HP infection (n = 1,995) by multivariate logistic regression analyses

Variable	ITP (n = 117)	Crude OR	*p*-value	Adjusted OR*	*p*-value
HPE					
Without	70 (8.7)	2.31 (1.58–3.38)	< 0.001	1.74 (1.14–2.65)	0.010
With	47 (4.0)	Reference		Reference	
Sex					
Male	85 (7.5)	2.07 (1.36–3.14)	0.001	2.23 (1.45–3.43)	< 0.001
Female	32 (3.8)	Reference		Reference	
Age subgroup					
Age < 65	62 (4.8)	Reference		Reference	
Age ≥ 65	55 (7.7)	1.64 (1.13–2.38)	0.010	1.45 (0.97–2.17)	0.070
Comorbidity					
Peptic ulcer disease	41 (4.4)	0.60 (0.41–0.89)	0.011	0.65 (0.42–1.01)	0.054
Hypertension	34 (6.6)	1.20 (0.79–1.81)	0.394	0.94 (0.58–1.51)	0.790
GERD	9 (2.87)	0.43 (0.22–0.86)	0.017	0.52 (0.26–1.04)	0.065
Diabetes	27 (7.4)	1.37 (0.88–2.14)	0.169	0.69 (0.38–1.23)	0.206
Stroke	7 (5.7)	0.97 (0.44–2.12)	0.934	0.56 (0.24–1.29)	0.172
Anemia	9 (17.7)	3.64 (1.73–7.68)	< 0.001	3.76 (1.69–8.38)	0.001

*ITP* Immune thrombocytopenia, *HP Helicobacter pylori*, *OR* Odds ratio, *GERD* Gastroesophageal reflux disease

*Adjusted for age, sex, the CCI score, and comorbidities, including peptic ulcer disease, hypertension, GERD, diabetes, stroke, and anemia

## Discussion

This study showed that patients without HPE had a higher risk of developing ITP than those with HPE, particularly males. In addition to HPE, the male sex and anemia were independent predictors of subsequent ITP in patients with HP infection. Moreover, patients without HPE also had a higher risk of severe thrombocytopenia with platelet counts < 30,000 per μL than those with HPE.

Immune reaction may explain the association between HP infection and ITP and the finding that HPE decreased the risk of ITP in this study. A study involving adult Japanese patients to investigate the role of molecular mimicry in chronic ITP found that there was HP cytotoxin-associated gene A (CagA) protein in the platelet eluates of the patients with chronic ITP and that HPE decreased the level of anti-CagA antibody [17]. They concluded that CagA contributes to the pathogenesis of ITP [17]. A basic study reported that the HP urease B antibody could cross-react with human platelet glycoprotein IIIa and may inhibit platelet aggregation [18]. This finding suggests that HP urease B is a cause of HP infection involved in the development of ITP [18]. Some studies reported that the interaction between HP infection and surface glycoproteins Ib/IX, von Willebrand factor, and membrane-associated lipoprotein may also be the mechanism of the development of ITP [19]. The aforementioned findings provide us with a promising direction for further investigation of the exact pathogenesis of HP infection-related ITP.

In the male population, the higher risk of ITP was statistically significant; however, the difference was insignificant in the female population in this study. Sex hormones may play a major role in this difference [20]. In ITP, the overall female-to-male ratio is 3–4 to 1, and young women in the third or fourth decade predominate [21]. These findings suggest that sex hormones and other immune diseases, including systemic lupus erythematosus and multiple sclerosis, are responsible for the development of ITP [21]. HP infection is a risk factor for ITP. Women have more risk factors for ITP than men, and therefore, HPE became less influential for the development of ITP. In the female population, the adjusted OR was 1.69 with 95% CI of 0.78–3.69, and the number of ITP cases was only 19. Therefore, another explanation is that the sample size in this study was not large enough to show statistical significance. This study found that male sex was an independent predictor of ITP in all adult patients with HP infection, which may be explained by anti-CagA antibody. A study included 525 participants in Iran reported that the prevalence of serum anti-CagA IgG was statistically higher in males than in females (48.6% vs. 31.6%; *p* = 0.046) [22]. A further study involving more patients is needed to clarify this issue.

Although no direct evidence that supports the novel findings in this study, an increasing number of studies showed that using HPE as the first-line treatment is beneficial for patients with ITP [5, 6, 23–25]. In Japan, a study involving 207 patients with chronic ITP with HP infection reported that the platelet count had a higher response rate in patients with HPE than in those without HPE (63% vs*.* 33%; *p* < 0.005) [6]. HPE was even effective in refractory cases of chronic ITP that were unresponsive to splenectomy [6]. Therefore, the authors suggested HPE as the first-line treatment in patients with ITP with HP infection [6]. In Korea, a multicenter and prospective phase-II study was conducted to evaluate the effectiveness of HPE as a first-line treatment in HP-positive patients with chronic ITP and moderate thrombocytopenia [5]. The results showed that the overall response rate was 19.2% at 4 weeks, 57.7% at 3 months, 65.4% at 6 months, 30.8% at 12 months, and 69.2% for the maximal response [5]. They concluded that HPE is an effective first-line treatment in this population [5]. These studies provided us with indirect evidence that HPE may be considered as early as possible to prevent ITP in patients with HP infections, particularly in those at a high risk of bleeding.

Compared with the patients with HPE, patients without HPE had higher CCI, more comorbidities, and older age in Taiwan. This finding is compatible with previous studies, suggesting that older patients with more comorbidities tended not to receive HPE [26]. The reason is that older people who have more comorbidities have difficulty to follow the treatment due to the presence of certain combined medications and declined physical function [26]. Another reason is that clinicians might be reluctant to treat very old patients for the concern of complication [26].

The major study strength is that we found that HPE may decrease the risk of subsequent ITP in patients with HP infection, which provides an important reference for developing prevention strategies in this population. The limitations were as follows. First, some data may have been missed because of the retrospective design of this study. Because of the missed data (particularly platelets), we excluded 2,366 patients from this study, which may have caused a selection bias. Second, the baseline age and CCI were different between patients with and without HPE, which may confound the results. However, we adjusted age and CCI by multivariate logistic regression analyses and found that HPE was associated with lower risk of developing ITP. Third, there was no data about test results for HP in patients after HPE in this study. Therefore, we could not investigate the effect if HP does not turn negative after treatment. Fourth, there was no data about cross-reactive antibodies against platelet antigens by molecular mimicry, which may help explain the pathogenesis of ITP. Fifth, the study size may not be large enough to show the true difference, which is particularly concerning in the stratified analysis in the female population. Sixth, the results are from a medical center in Taiwan, and therefore, its generalization needs external validation in other hospitals or nations. Further studies with more patients, prospective design, data about test results for HP in patients after HPE, and cross-reactive antibodies against platelet antigens may also be warranted.

## Conclusion

This is the first cohort study to delineate that adult patient with HP infection not receiving HPE had a higher risk of developing ITP. The possible reason is that HPE may decrease the immune reaction caused by HP infection. The decreased risk was found only in the male population, which may be associated with differences in sex hormones and other immune diseases between the two sexes. Another reason is that the sample size was not large enough to show a statistical difference. We suggest that HPE should be considered in patients with HPE to prevent ITP, particularly in males and those who have a history of anemia. Further studies involving more patients that adopt a prospective study design with external validations from other hospitals and nations are warranted.

## Supplementary Information

**Additional file 1: Table S1.** Comparison of the risk of having platelet counts < 30,000 per μL between adult patients without and with HPE by multivariate logistic regression analyses. **Table S2.** Comparison of HPE for developing ITP in adult patients with platelet count between 100,000 and 150,000 per μL by univariate analyses.

## Data Availability

The datasets generated during and/or analyzed during this study are available from the corresponding author on reasonable request.

## Abbreviations

HP: *Helicobacter pylori*
HPE: *Helicobacter pylori* eradication therapy
ITP: Immune thrombocytopenia
CCI: Charlson comorbidity index
OR: Odds ratio
CI: Confidence interval
CMMC: Chi Mei Medical Center
μL: Microliter
GERD: Gastroesophageal reflux disease
CAD: Coronary artery disease
IRB: Institutional review board
SD: Standard deviation
CagA: Cytotoxic-associated gene A
