# Supplementary material for: Eradication therapy may decrease the risk of immune thrombocytopenia after Helicobacter pylori infection: a retrospective cohort study in Taiwan
Source: BMC Gastroenterol. 2023 Feb 8;23:36. doi: 10.1186/s12876-023-02664-z (PMC9907885; doi:10.1186/s12876-023-02664-z)
Supplement: Supplementary file 1 — Additional file 1: Table S1. Comparison of the risk of having platelet counts < 30,000 per μL between adult patients without and with HPE by multivariate logistic regression analyses. Table S2. Comparison of HPE for developing ITP in adult patients with platelet count between 100,000 and 150,000 per μL by univariate analyses. [file 12876_2023_2664_MOESM1_ESM.docx]

**Table S1.** Comparison of the risk of having platelet counts < 30,000 per μL between adult patients without and with HPE by multivariate logistic regression analyses

| Without HPE vs. With HPE (reference) | Patients | Platelet < 30,000 per μL | Adjusted OR* | *P*-value |
| --- | --- | --- | --- | --- |
| Overall analysis | 807 | 11 (1.4) | 8.46 (1.74−41.16) | 0.008 |
| Stratified analysis |  |  |  |  |
| Sex |  |  |  |  |
| Male | 476 | 7 (1.5) | 4.40 (0.78−24.69) | 0.093 |
| Female | 331 | 4 (1.2) | >999.99 (<0.001–>999.99) | 0.935 |
|  |  |  |  |  |
| Age subgroup |  |  |  |  |
| Age < 65 | 471 | 4 (0.9) | 4.48 (0.39−50.95) | 0.227 |
| Age ≥ 65 | 336 | 7 (2.1) | 10.38 (1.21−89.18) | 0.033 |
| Comorbidity |  |  |  |  |
| GERD | 84 | 0 (0) | <0.001 (<0.001−>999.99) | 0.941 |
| Peptic ulcer disease | 224 | 3 (1.3) | 4.73 (0.75−29.98) | 0.099 |
| Hypertension | 218 | 3 (1.4) | >999.99 (<0.001−>999.99) | 0.902 |
| Diabetes | 188 | 3 (1.6) | 4.81 (0.39−59.61) | 0.221 |
| Stroke | 74 | 2 (2.7) | >999.99 (<0.001−>999.99) | 0.951 |

*Adjusted for age, sex, the CCI score, and comorbidities, including peptic ulcer disease, hypertension, GERD, diabetes, stroke, and anemia. HPE, *Helicobacter pylori* eradication therapy; OR, odds ratio; CCI, Charlson Comorbidity Index; GERD, gastroesophageal reflux disease.

**Table S2.** Comparison of HPE for developing ITP in adult patients with platelet count between 100,000 and 150,000 per μL by univariate analyses

|  | Overall  (n = 56) | With HPE  (n = 28) | Without HPE  (n = 28) | *P*-value |
| --- | --- | --- | --- | --- |
| Platelet before HP (+) |  |  |  |  |
| Mean ± SD | 128.8 ± 14.4 | 130.2 ± 14.7 | 127.5 ± 14.3 | 0.480 |
| Median (Q1-Q3) | 131.0 (115.0−142.0) | 133.0 (115.0−143.0) | 127.5 (115.0−140.5) | 0.446 |
| Platelet after HP (+) |  |  |  |  |
| Mean ± SD | 136.5 ± 68.0 | 133.1 ± 57.8 | 139.9 ± 77.7 | 0.712 |
| Median (Q1−Q3) | 127.0 (94.5−177.0) | 121.5 (97.0−161.5) | 134.0 (82.5−183.5) | 0.774 |
| ITP, n (%) | 17 | 8 (47.1) | 9 (52.9) | 0.771 |
| Platelet after HP (+) |  |  |  |  |
| Mean ± SD | 64.7 ± 27.3 | 71.6 ± 27.1 | 58.6 ± 27.5 | 0.340 |
| Median (Q1−Q3) | 64.0 (35.0−89.0) | 79.0 (47.5−94.5) | 61.0 (32.0−80.0) | 0.268 |

HPE, *Helicobacter pylori* eradication therapy; ITP, Immune thrombocytopenia; HP, *Helicobacter pylori*; SD, standard deviation.
